# Glioblastoma Therapy with Cytotoxic Mesenchymal Stromal Cells Optimized by Bioluminescence Imaging of Tumor and Therapeutic Cell Response

**DOI:** 10.1371/journal.pone.0035148

**Published:** 2012-04-17

**Authors:** Maria Alieva, Juli R. Bagó, Elisabet Aguilar, Carolina Soler-Botija, Olaia F. Vila, Joan Molet, Sanjiv S. Gambhir, Nuria Rubio, Jerónimo Blanco

**Affiliations:** 1 Cardiovascular Research Center, CSIC-ICCC, CIBER-BBN, Barcelona, Spain; 2 Cardiology Service, Hospital Universitari Germans Trias i Pujol, Badalona, Spain; 3 Neurosurgery Unit, Hospital Santa Creu i Sant Pau, Barcelona, Spain; 4 Department of Radiology, The Bio-X Program, Stanford University, Stanford, California, United States of America; University of Michigan School of Medicine, United States of America

## Abstract

Genetically modified adipose tissue derived mesenchymal stromal cells (hAMSCs) with tumor homing capacity have been proposed for localized therapy of chemo- and radiotherapy resistant glioblastomas. We demonstrate an effective procedure to optimize glioblastoma therapy based on the use of genetically modified hAMSCs and *in vivo* non invasive monitoring of tumor and therapeutic cells. Glioblastoma U87 cells expressing *Photinus pyralis* luciferase (Pluc) were implanted in combination with hAMSCs expressing a trifunctional *Renilla reniformis* luciferase-red fluorescent protein-thymidine kinase reporter in the brains of SCID mice that were subsequently treated with ganciclovir (GCV). The resulting optimized therapy was effective and monitoring of tumor cells by bioluminescence imaging (BLI) showed that after 49 days GCV treatment reduced significantly the hAMSC treated tumors; by a factor of 10^4^ relative to controls. Using a Pluc reporter regulated by an endothelial specific promoter and *in vivo* BLI to image hAMSC differentiation we gained insight on the therapeutic mechanism. Implanted hAMSCs homed to tumor vessels, where they differentiated to endothelial cells. We propose that the tumor killing efficiency of genetically modified hAMSCs results from their association with the tumor vascular system and should be useful vehicles to deliver localized therapy to glioblastoma surgical borders following tumor resection.

## Introduction

Glioblastoma multiforme (GBM), a brain tumor with high morbidity and mortality, accounts for the highest proportion of deaths from brain tumors [1]. The average survival of patients diagnosed with GBM is 14 months, and only 2% of the patients survive up to 3 years [2], [3]. Current treatment protocols for GBM are ineffective due to its invasiveness, which precludes complete surgical removal and to persistence of radiation and chemotherapy resistant cells after therapy. A promising approach based on the use of cellular vehicles to deliver localized treatment targeting the primary tumor mass has been proposed [4]. Different types of stem cells have shown therapeutic potential against glioblastomas [5], [6], [7], [8]. However, due to their tumor homing capacity and ease of genetic manipulation bone marrow mesenchymal stromal cells appear as particularly promising therapeutic vehicles [9]. Human mesenchymal stromal cells are abundant and easily extracted from adipose tissue and have properties similar to those of their bone marrow counterparts: ease of expansion from autologous tissue [10], [11], [12], demonstrated homing capacity towards tumors [13], [14], [15] and participation in the stroma of different types of tumors [16], ease of genetic manipulation [17], capacity to inhibit immune and inflammatory reactions [18] and genetic stability, not generating tumors when implanted during long periods of time [19]. Recently, a rat C6 glioblastoma tumor model was successfully treated with human adipose tissue mesenchymal stromal cells (hAMSCs) modified to express cytosine deaminase:uracil phosphoribosyltransferase [20].

Targeted cytotoxicity strategies, developed during the last 20 years for gene therapy, are based on the activation of a systemically delivered low toxicity prodrug to a cytotoxic agent in the vicinity of tumors, mediated by the action of a specific activating enzyme previously introduced in the therapy vehicle [21]. The delivery of cytotoxicity in the vicinity of a tumor has the advantage of avoiding systemic toxicity. The thymidine kinase encoded by herpes simplex virus (HSV-TK) was used in the first cell suicide gene therapy proof of principle and still is one of the most widely used systems in clinical and experimental applications [22], [23]. This cytotoxic system has been applied to the treatment of gliomas with significant increases in survival [24]. HSV-TK is responsible for the conversion of deoxythimidine to deoxythyimidine monophosphate in infected cells. Due to its relaxed specificity, this enzyme can phosphorylate a variety of nucleotide analogs including ganciclovir (GCV), a 2′-deoxiguanosine analog [25]. The incorporation of phosphorylated GCV in DNA results in DNA polymerase inhibition, chain termination and cell suicide [2], [13], [14], [26]. Transfer of dying cell components through gap junctions and diffusion results in the killing of neighboring cells, with amplification of the therapeutic effect.

In the current work, we develop and demonstrate an efficient therapeutic strategy model against a *Photynus pyralis* luciferase (Pluc) and enhanced green protein (eGFP) expressing U87 glioblastoma model in SCID mice, using hAMSCs genetically modified to express a tri-functional reporter comprising *Renilla reniformis* luciferase (Rluc), red fluorescent protein (RFP) and a truncated version of HSV-TK (tTK). The latter enzyme has a 30 fold higher activity than wild type HSV-TK [26], [27]. By taking advantage of bioluminescence imaging (BLI) to noninvasively monitor luciferase expressing tumor and therapeutic cells, we were able to fine tune and evaluate the therapeutic process in real time. In addition, the imaging platform allowed us to observe the behavior of therapeutic hAMSCs, offering insight into the therapeutic mechanism.

## Results

### Photoprotein expressing hAMSC and U87 cells

To noninvasively monitor the behavior of tumor and therapeutic cells during therapy, hAMSCs were genetically modified by transduction with a lentiviral vectors for stable expression of a tri-functional chimeric reporter including Rluc, RFP and tTK activities, to generate Rluc-R-tTK-hAMSC or “therapeutic vehicles". Human glioblastoma U87 tumor cells were also genetically modified by transduction with a lentiviral vector for stable expression of a chimeric reporter with Pluc and eGFP activities, to generate “Pluc-G-U87" tumor cells. This strategy allowed independent imaging of therapeutic and tumor cells using non-cross-reacting luciferase substrates, coelenterazin and luciferin, respectively. In addition, the inclusion of two different fluorescent protein reporters, allowed selection of labeled cells by FACS, and detection of therapeutic and tumor cells by fluorescence confocal microscopy of tissue sections. Lentiviral transduction did not change doubling time or immunophenotype of Rluc-R-tTK-hAMSCs, as determined by flow cytometry analysis of hAMSC markers (Fig. S1).

### Bystander killing of Pluc-G-U87 glioblastoma cells by Rluc-R-tTK-hAMSC *in vitro*

**Figure 1:**
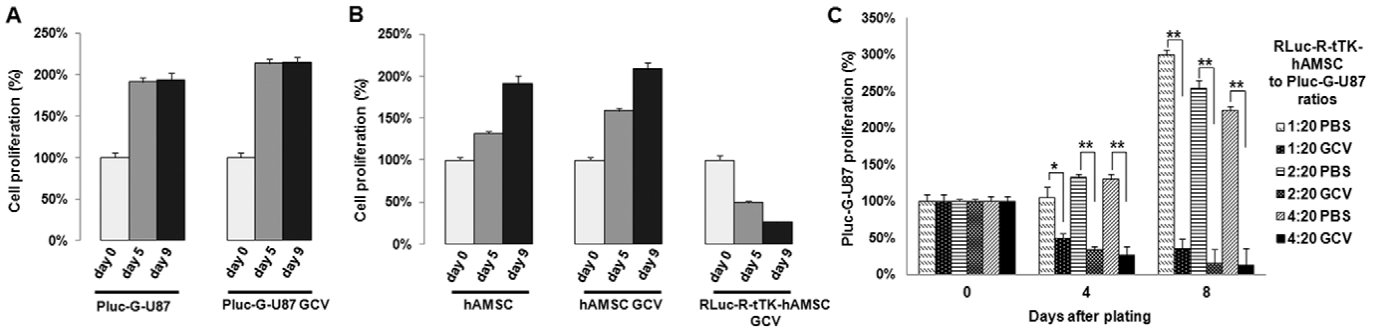
Sensitivity of PLuc-G-U87 cells, Rluc-R-tTK-hAMSC and hAMSC and co-cultures comprising different proportions of Rluc-R-tTK-hAMSCs and PLuc-U87 cells to GCV. Cells were grown during the indicated times and treated with either GCV (4 µg/ml) or PBS, as indicated. Number of viable cells was evaluated spectrophotometrically by standard 3-(4-5-dimethyl-2-yl)-5-(3-carboxymethoxyphenyl)-2-(4-sulfophenyl)-2H-tetrazolium salt (MTS assay) (**A, B**), or by BLI (**C**), and expressed as percentage increase relative to cell number at day 0. Histograms show mean ± SEM, *p<0.05, **p<0.01 n = 4 for each group.

To demonstrate tTK gene was functional in the genetically modified therapeutic vehicles, replicate cultures of Pluc-G-U87, hAMSCs and Rluc-R-tTK-hAMSCs seeded in tissue culture plates were treated with ganciclovir (GCV) or PBS and cell survival assessed using the CellTiter 96 Aqueous One Solution Cell Proliferation Assay. Treatment with GCV had no effect on survival of Pluc-G-U87 alone (Fig. 1A) or on wt hAMSCs lacking the tTK gene (Fig. 1B). What is more, in the absence of GCV the growth pattern of hAMSCs was similar regardless of whether they expressed or not the tTK gene (Fig. S1B), indicating that the tTK gene had no negative effect on cell proliferation. However, as expected, GCV treatment did exert a strong cytotoxic effect, proportional to its concentration, on tTK expressing hAMSCs (Fig. S2) (*P<0,05).

In a different experiment to assess bystander effect, cocultures of luciferase expressing therapeutic and tumor cells in different proportions (1∶20, 2∶20, 4∶20) Rluc-R-tTK-hAMSC∶Pluc-G-U87 were treated with GCV (4 µg/ml) or PBS. In these experiments, where tumor and therapeutic cells express luciferase reporters, survival of Pluc-G-U87 cells was determined by bioluminescence.

As shown in Fig. 1C, viability of Pluc-G-U87 cells in the presence of GCV was inversely proportional to the ratio of therapeutic to tumor cells (88%, 93%, 94% killing for 1∶20, 2∶20, 4∶20 Rluc-R-tTK-hAMSC∶Pluc-G-U87), but was not affected by treatment with PBS (*p<0,05; **p>0,01).

### Detection sensitivity of Pluc-G-U87 and Rluc-R-tTK-hAMSC *in vivo*

**Figure 2:**
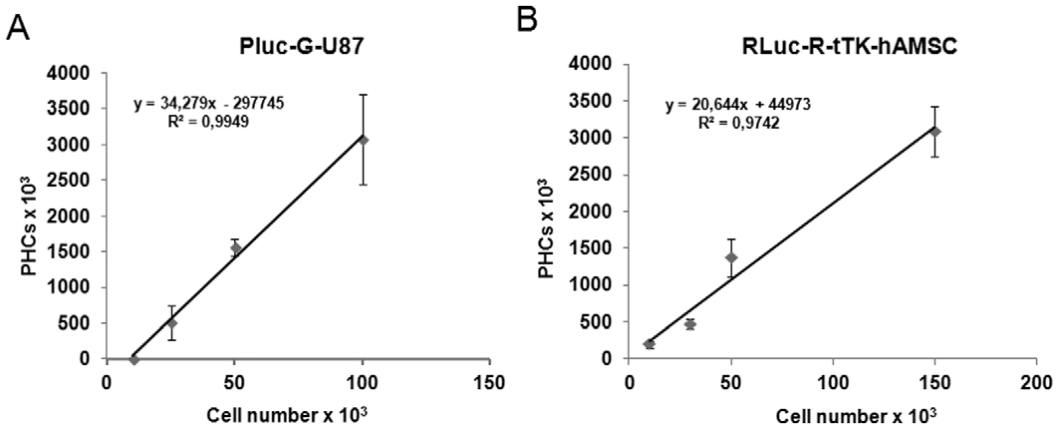
*In vivo* BLI sensitivity. The indicated numbers of Pluc-G-U87 (**A**) and Rluc-R-tTK-hAMSC cells (**B**) were stereotactically implanted, at the same coordinates later used for experiments in the brain of immunosupressed mice and imaged after luciferin or coelenterazine substrate administration, respectively. The number of light events (PHCs) recorded from the mice brains was plotted, after background subtraction, versus the number of implanted cells. R^2^ correlation coefficient. Data are presented as mean ±SEM, n = 4 in each group.

Skull bone and intervening brain tissue in live animals reduce the efficiency with which light photons reach the video camera detector used for BLI. To establish a correlation between light measurements and cell number, predetermined numbers of Pluc-G-U87 and Rluc-R-tTK-hAMSC were implanted in the brain of live mice at specific coordinates (0,6 mm posterior, 2 mm lateral and 2,75 mm depth respect Bregma) later used for experiments, and immediately after implantation imaged by BLI after administration of the corresponding luciferase substrate, luciferin or coelenterazine. The number of recorded photon events (PHCs) was extracted from recorded images and plotted, after background subtraction, versus the number of inoculated cells. As shown in Fig. 2, detection sensitivity (slope of the correlation curve) was similar for both cell types and there was a good linear correlation between the amount of light produced and the number of cells implanted, 34,2 PHCs/cell, R^2^ = 0,994 and 20,64 PHCs/cell, R^2^ = 0,974 for Pluc-G-U87 and Rluc-R-tTK-hAMSC, respectively.

### Optimal Rluc-R-tTK-hAMSC dose for *in vivo* bystander effect

**Figure 3:**
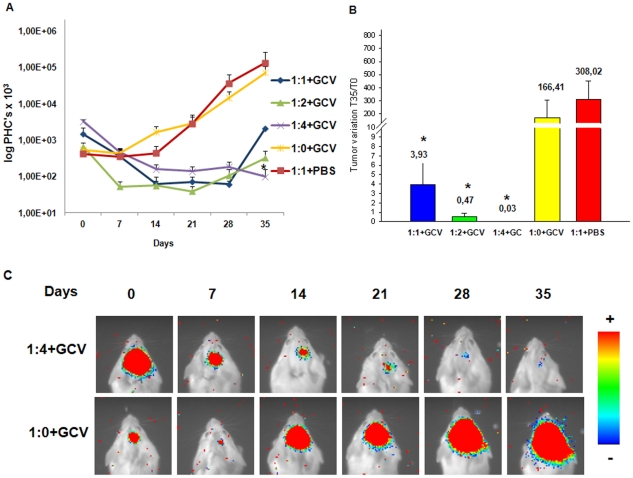
*In vivo* optimization of tumor to therapeutic cell ratio. Tumor and therapeutic cells mixed in proportions 1∶0, 1∶1, 1∶2 and 1∶4 (Pluc-G-U87∶Rluc-R-tTK-hAMSC) were implanted in the brain of SCID mice and treated at day 6 i.p. with either GCV or PBS as indicated and monitored by BLI at weekly intervals. (**A**) The graph shows *in vivo* changes in light production by Pluc expressing tumor cells resulting from GCV treatment. (**B**) Histogram showing PHC values at the end of the experiment (T = 35). Values were normalized relative to those at T = 0 according to the formula [(T5/T0)×100 = tumor variation (%)]. Data are shown as mean ± SEM, *P<0.05, compared to the 1∶0+GCV group, n = 5 for each group. (**C**) Representative pseudo-color BLI images showing evolution of tumor size during treatment of animals inoculated with the indicated proportions of Pluc-G-U87 to Rluc-R-tTK-hAMSC. Color bars represent light intensity levels from Pluc luciferase, ranging from low: blue to high: red. Luciferase images are superimposed on black and white with images of the same mouse.

Mixed cell populations comprising Pluc-G-U87 cells (4×10^4^) and different proportions of Rluc-R-tTK-hAMSCs, (1∶1, 1∶2, 1∶4) respectively, were injected in the brain of SCID mice at the previously indicated coordinates (n = 15) to establish the best proportion of therapeutic to tumor cells. Two control groups (n = 10), were also included, one of them consisting of mice injected with 4×10^4^ Pluc-G-U87 cells only, that was treated with GCV; and the other consisting of mice injected with the same number of Pluc-G-U87 cells in a 1∶1 proportion with Rluc-R-tTK-hAMSCs, that was treated with PBS. Imaging of Rluc activity was used to monitor implantation of Rluc-R-tTK-hAMSCs. Beginning six days after the cell implantation (T = 0), and during a 35 day period, the inoculated mice were treated daily by i.p. administration of GCV or PBS, as indicated. Light emission by Pluc luciferase was monitored by BLI from the beginning of GCV treatment and every week thereafter. Image sequences of tumors were used to monitor tumor growth during the experiment. Analysis of photons captured in all images and statistical evaluation showed (Fig. 3A and B) that mixed cell tumors (1∶1) treated with PBS grew at a similar rate than control tumors treated with GCV but without therapeutic cells (p>0,05). However, treatment with GCV of tumors bearing therapeutic cells inhibited tumor growth relative to controls. Although a trend in tumor growth inhibition appears from the start of the GCV treatment, a clear inhibition of tumor growth was apparent by day 21 and was evident by day 35 of GCV treatment in all the therapeutic cell proportions tested. The therapeutic effect directly correlated with the proportion of therapeutic cells used, being 1∶4 the most effective cell dose, and in this group of mice, by the end of the experiment (T = 35) the amount of light produced by the tumors was 0,14% of the light produced by those in the control group (Pluc-G-U87 only) (p<0,05). Analysis comparing tumor size at the start (T = 0) and end (T = 35) of treatments shows a significant size reduction (p<0,05) for all the tumors treated with therapeutic cells plus GCV, but not for controls where either GCV or therapeutic cells were omitted. In the 1∶4+GCV group, only 3% of light produced by tumor cells at T = 0 persisted at the inoculation sites following GCV treatment. Fig. 3C shows representative images from single animals from the groups (n = 5) treated with GCV and with or without therapeutic cells (1∶4+GCV).

### *In vivo* Rluc-R-tTK-hAMSC mediated bystander glioblastoma therapy

**Figure 4:**
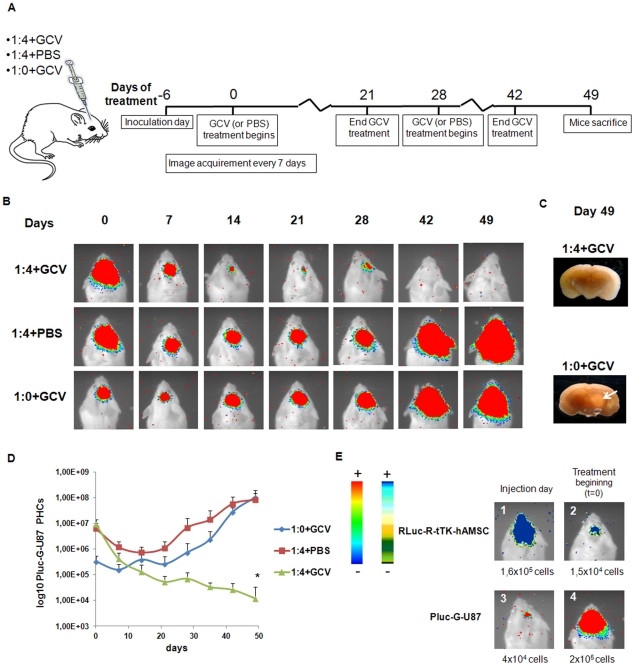
*In vivo* BLI of hAMSC mediated tumor therapy. (**A**) Diagram illustrating experimental procedure. (**B**) Composite pseudo-color BLI images from mice implanted with 4×10^4^ Pluc-G-U87 plus 1.6×10^5^ Rluc-R-tTK-hAMSCs and treated with GCV (1∶4+GCV); 4×10^4^ Pluc-G-U87 plus 1.6×10^5^ Rluc-R-tTK-hAMSC and treated with PBS (1∶0+GCV), and with 4×10^4^ Pluc-G-U87 only and treated with GCV. Images were acquired once per week. (**C**) Representative images of brain sections at day 49. Top: brain of a 1∶4+GCV treated mouse. Bottom: brain of a 1∶0+GCV mouse, arrow points to tumor area. (**D**) *In vivo* changes in light production by U87 tumors expressing Pluc, resulting from GCV treatment and tumor cell death. Light values, calculated from the recorded images, are represented as log10 mean ± SEM; n = 8; * p<0.05, compared to the 1∶0+GCV. (**E**) Representative pseudo-color images from Rluc-R-tTK-hAMSC and Pluc-G-U87 at day of implantation, T = −6 (1 and 3) and at initiation of GCV treatment, T = 0 (2 and 4). Luciferase images are superimposed on black and white images of the same mouse. The included numerical values indicate the cell numbers extrapolated from the “light vs cells" standard curve. Arbitrary color bars illustrate relative light intensities from PLuc and Rluc luciferases, lowest: blue and black; highest: red and blue, respectively.

A group of 24, 6 week old SCID mice was stereotactically inoculated at the previously indicated coordinates with either a mixture of 4×10^4^ Pluc-G-U87 cells and 1.6×10^5^ Rluc-R-tTK-hAMSCs (n = 16) or with 4×10^4^ Pluc-G-U87 tumor cells only (n = 8). Six days post implantation (T = 0), the mice were imaged by BLI to monitor Pluc activity from the Pluc-G-U87 tumors and Rluc activity from Rluc-R-tTK-hAMSC cells, and a treatment regime was initiated as described in the diagram of Fig. 4A. In brief, one half of the mice implanted with Pluc-G-U87 and Rluc-R-tTK-hAMSC cells received GCV, while the other half received the same volume of PBS. Mice implanted with only Pluc-G-U87 cells also received GCV. Treatment was administered during two 3 week periods, separated by a one week rest period. At day 49 all the control mice were sacrificed, as required by the animal welfare protocol.

Bioluminescence imaging (BLI) was used to monitor tumor development during the experiment. As indicated in Fig. 4B, showing representative images from single animals in each of the experimental groups, treatment of mixed cell tumors with PBS, or of control tumors (lacking therapeutic cells) with GCV had no inhibitory effect on tumor growth, and light production by Pluc-G-U87 cells progressively increased up to the end of the experiment. However, treatment of mixed cell tumors with GCV resulted in the progressive reduction of light produced by Pluc-G-U87 cells up to the end of the experiment, when light from Pluc-G-U87 cells reached background level (Fig S3). Quantitative evaluation of therapeutic effect is shown by plots of total light events recorded in images from the tumor areas vs. time (Fig. 4D). This analysis shows that the amount of light produced by tumors containing therapeutic cells that were treated with GCV, by the end of the experiment was 10^4^ times lower than that produced by control tumors treated with GCV or by tumors with therapeutic cells treated with PBS (p<0,05). Moreover, by the end of the experiment, light produced by tumors treated with therapeutic cells plus GCV was only 0,12% the amount of light produced by the same tumors at T = 0 (6 days after cell implantation) (n = 8). Cell killing detected by BLI could also be verified at the macroscopic level by visual comparison of brain sections from control mice and those treated with therapeutic cells plus GCV (Fig. 4C), and while sections from control mice brains showed a clear tumor mass, those from mice inoculated with tumor plus therapeutic cells and treated with GCV had a normal macroscopic appearance and showed no signs of tumors.

**Figure 5:**
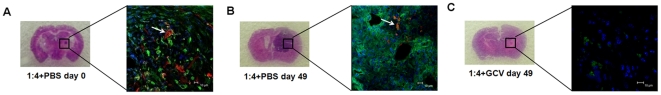
Effect of GCV on fluorescent Rluc-R-tTK-hAMSC at tumor implantation sites. Left, representative images of HE stained brain sections; Right, representative fluorescence confocal microscope images showing implanted red fluorescent cells Rluc-R-tTK-hAMSC (arrow), and Pluc-G-U87 cells (green). (**A**) Control mouse (1∶4+PBS) at T = 0, (**B**) control mouse (1∶4+PBS) at T = 49, (**C**) treated mouse (1∶4+GCV) at day 49. Blue, Hoescht stained nuclei. Scale bar = 10 µm.

Corroboration of BLI analysis was also provided by fluorescence confocal microscope observation of mouse brain sections that showed (Fig. 5) the presence of red fluorescent Rluc-R-tTK-hAMSCs evenly distributed throughout the tumor implantation site mixed with green fluorescent tumor cells, at T = 0 before starting GCV treatment (Fig. 5A). In well developed tumors (T = 49) from control group 1∶4+PBS, red fluorescent Rluc-R-tTK-hAMSCs could also be detected in clusters within the green fluorescent mass of the well developed tumor (Fig. 5B). However, brains from mice in the 1∶4+GCV group had few detectable tumor cells and no detectable Rluc-R-tTK-hAMSCs, in accordance with the BLI analysis (Fig. 5C).

### Post-engraftment survival of therapeutic hAMSCs

The fate of Rluc-R-tTK-hAMSCs was monitored by BLI at the implantation day (T = −6) and 6 days later (T = 0), at the beginning of GCV treatment (Fig. 4E). The number of detected PHCs at T = 0 corresponded with that produced by 1.5×10^4^ Rluc-R-tTK-hAMSCs in standard plots (Fig. 2B), near to a 10 fold decrease relative to T = −6. However, due to their rapid proliferation the amount of light produced by Pluc-G-U87 tumor cells in the 1∶4+GCV and 1∶4+PBS groups, at T = 0 corresponded to approximately 2×10^5^ cells (Fig. 4E). Therefore, during the 6 days previous to GCV treatment, the ratio of PLuc-G-U87: Rluc-R-tTK-hAMSCs changed by 52 fold, from 1∶4 to 13∶1. Thus, the later is the actual ratio at which therapeutic effect is exerted.

### Fate of tumor implanted Rluc-R-tTK-hAMSCs therapeutic cells

**Figure 6:**
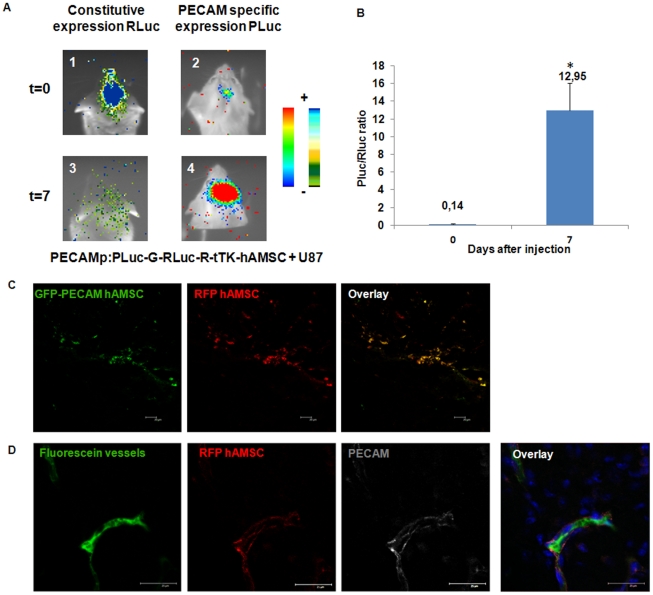
Endothelial differentiation of hAMSCs in U87 tumors. Double transduced hAMSCs expressing PECAM-promoter regulated PLuc-GFP and CMV-promoter regulated Rluc-R-tTK were mixed 4∶1 with unlabelled U87 glioma cells, implanted in the brain of mice and imaged at the indicated times. Mice were then inoculated with a 2,000,000 MW FITC-conjugated dextran and the brains harvested, fixed in formalin and prepared for microscopy. (**A**) Representative BLI images showing Rluc (left) and Pluc (right) activities at T = 0 (1 and 2) and T = 7 (3 and 4), respectively. Pseudo color images are superimposed on black and white dorsal images of recipient mice. Color bars illustrate relative light intensities of Pluc (right) and Rluc (left); low: blue and black; high: red and blue, respectively. (**B**) Histograms showing the change in the ratio of Pluc/Rluc activities during the indicated time period. Bars represent mean ± SEM of photon counts recorded in BLI images. * p<0.05, n = 4. (**C**) Representative laser confocal microscope images of the corresponding brain tumor slices showing hAMSCs expressing CMV-promoter regulated RFP (red) and PECAM-promoter regulated eGFP (green). Scale bar = 20 µm. (**D**) FITC-stained microvessel (green), associated with red fluorescent hAMSCs (red), also labeled by an anti human PECAM antibody (gray) Scale bar = 25 µm.

In an attempt to understand the mechanism by which the Rluc-R-tTK-hAMSCs+GCV treatment achieves the observed high degree of therapeutic effect, the differentiation behavior of therapeutic cells was analyzed. To do this, we isolated by FACS Rluc-R-tTK-hAMSCs and labeled them with a second Pluc-eGFP reporter under transcriptional control by the PECAM/CD31 promoter, as a reporter of differentiation to the endothelial lineage. With this strategy, changes in PECAM-regulated luciferase expression could be quantitatively related to those of the constitutively expressed Rluc, now an internal standard in the same cell that allows the elimination of potential artifacts related to changes in hAMSC number. The doubly labeled hAMSCs were mixed with un-labeled U87 tumor cells and implanted at the standard brain location. BLI analysis of Pluc and Rluc expression at days 0 and 7 post-implantation showed (Fig. 6A) that while the bulk of implanted hAMSCs disappears from the tumor during the 7 day period, cells expressing PECAM-promoter regulated Pluc remained associated to the tumor. Moreover, the ratio of PECAM/CD31 promoter-regulated Pluc activity to constitutively-expressed Rluc increased considerably by 92 fold, indicating that a subpopulation of the implanted hAMSCs had differentiated to the endothelial lineage and remained at the tumor site (Fig. 6B). In addition, analysis by laser confocal microscopy of tumor sections showed (Fig. 6C) that implanted cells expressing the RFP and eGFP regulated by the PECAM/CD31 promoter were closely associated to tumor microvascular structures and had endothelial morphology. The endothelial lineage of therapeutic cells association to vascular structures in the tumor was further verified by perfusing some of the mice with a fluorescein-conjugated high MW dextran not diffusible past the vascular endothelium. Confocal microscope analysis of sections of the perfused tumors showed the presence of RFP expressing therapeutic cells that positively stained with an anti-human PECAM/CD31 antibody and were closely associated to FITC-dextran labeled vascular structures (Fig. 6D).

In this experiment all the implanted therapeutic cells express RFP but only a fraction of them is induced to express the endothelial lineage reporters. This double label strategy offered the possibility of determining if the decrease in RLuc expressing therapeutic cells previously observed in tumors could be due to silencing of the CMV promoter that regulates RLuc, RFP and tTK expression. To do this, tumor sections were also analyzed to determine the proportion of eGFP expressing therapeutic cells that did not express RFP. Our assessment showed that most of eGFP expressing cells also expressed RFP and only 10% or less of the eGFP expressing cells did not express RFP, ruling out CMV promoter silencing as the cause for the decrease in production RLuc. photons within tumors.

### Implantation of Rluc-R-tTK-hAMSC directly on established tumors also mediates bystander glioblastoma therapy

To verify that the Rluc-R-tTK-hAMSC mediated therapeutic approach is also effective under more clinical like conditions, 6 week old SCID mice were first stereotactically inoculated at the previously indicated coordinates only with 4×10^4^ Pluc-G-U87 cells. Six days post implantation (T = 0) one halve of the mice randomly chosen (n = 4) received an intracranial intratumoral injection of 1.6×10^5^ Rluc-R-tTK-hAMSCs. On day three mice were imaged by BLI to monitor Pluc-G-U87 tumor development, following which GCV treatment was initiated consistent of a daily dose of .GCV during a four week periods followed by 3 day rest periods. Weekly BLI was used to monitor tumor development during the experiment. On days 33 and 68, when the drop in Renilla luciferase emission indicated the disappearance of implanted Rluc-R-tTK-hAMSCs, additional intratumoral inoculations of 1.6×10^5^ Rluc-R-tTK-hAMSCs were performed.

**Figure 7:**
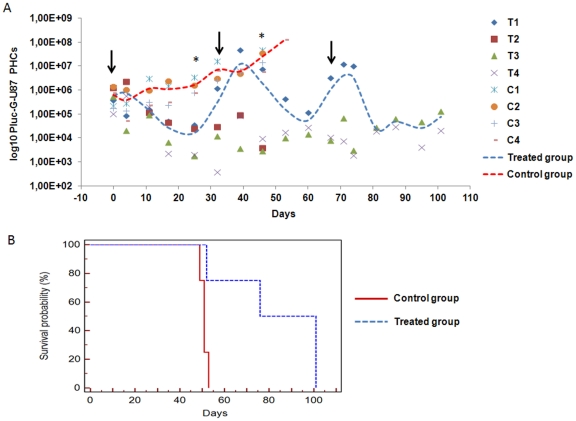
Response of U87 tumors to direct implantation of therapeutic cells and GCV treatment. (**A**) *In vivo* changes in light production by U87 tumors expressing Pluc, resulting from inoculation of Rluc-R-tTK-hAMSCs and GCV treatment. Dots represent PHCs values from individual animals (T: Treated mice; C: Control mice). The bars represent the average values from each group. Arrows indicate the inoculation of Rluc-R-tTK-hAMSCs. Light values, calculated from the recorded images, are represented as log10 of the mean ± SEM; n = 4; * p<0.05, compared to the control group. (**B**) Kaplan-Meyer graph sumarizing mice survival following the above treatment. Median survival, treated mice, 88,5 d; control mice, 51 d, p<0.05.

As shown in Fig. 7A, the administration of Rluc-R-tTK-hAMSCs in combination with GCV results in the inhibition of tumor growth. Moreover, the tendency of treated tumors to regain growth when implanted therapeutic cells disappear is effectively conteracted by repeated inoculation of additional Rluc-R-tTK-hAMSC and GCV treatment. By day 53 right before all the control mice had died, light produced by tumors treated with therapeutic cells plus GCV was only 0,12% of that produced by the control tumors. Kaplan-Meier survival graph (Fig. 7B) showed that the treatment protocol resulted in significant (p<0.05) increase in animal survival. Median survival was 88,5 d (treated animals) and 51 d (control animals).

## Discussion

In the current work we demonstrate a procedure to develop therapeutic strategies against a human U87 glioblastomas and an effective therapy model in SCID mice. The procedure is based on the use of hAMSCs genetically modified to express the herpes Simplex tTK as vehicles to deliver bystander toxicity to tumors. To analyze the behavior of tumor and therapeutic cells, we labeled the former with a CMV promoter regulated Pluc-eGFP chimerical reporter, and the latter with a different trifunctional chimerical reporter comprising Rluc-RFP and tTK activities [27]. This approach allowed us to monitor the location and number of tumor and therapeutic cells in live mice during treatment with GCV, and optimize cell and prodrug treatment.

We established that both, tTK activity in genetically modified hAMSCs, and administration of GCV were both required for bystander killing of tumor cells co-cultivated with hAMSCs.

*In vivo* BLI of predetermined numbers of hAMSC and U87 cells, stereotactically implanted at a specific site of the mouse brain, allowed us to correlate the number of photon events recorded by BLI with that of implanted cells, for their quantification during therapy.

An optimum therapeutic ratio of 4 hAMSCs to 1 U87 cell was empirically determined by implanting predetermined proportions of both cell types in mouse brains and administering GCV i.p. after a 6 day period, allowed for tumor establishment. Analysis of hAMSC behavior during the 6 day pre-treatment period indicated that a large proportion of the therapeutic cells disappeared, while tumor cells proliferated. This lead to a 50 fold, reduction in the proportion of therapeutic to tumor cells, that was 1∶13 by the time the GCV treatment began. This result not only emphasizes the effectiveness of hAMSCs as therapeutic vehicles, but also suggests that procedures or scaffold materials for their protection would likely improve even more the therapeutic outcome. The disappearance of a large proportion of hAMSCs when injected in immunosupressed mice has been previously observed [28]. However, the reduction in hAMSC number should not be attributed to CMV promoter silencing. In a previous study by Vilalta et al. [19], hAMSCs expressing Pluc regulated by the constitutively active SV40 promoter also lost between 75–90% of cells within the first 10 days post inoculation in SCID mice. Moreover, since in our cell differentiation experiment all the therapeutic cells are initially red fluorescent and become also green fluorescent upon differentiation to the endothelial lineage, silencing of the CMV promoter should result in the appearance of green fluorescent cells that show no red fluorescence. Thus CMV silencing can be evaluated by determining the proportion of therapeutic cells expressing GFP (regulated by the inducible PECAM/CD31 promoter) that do not express RFP (regulated by the CMV promoter) in histological sections of tumors. While extensive silencing of the CMV promoter would be required to produce the 92 fold increase in PECAM/CD31-PLuc expression observed in the BLI experiments, our assessment found less than 10% of GFP positive cells that were not RFP positive. This result excludes CMV promoter silencing, and pointing to cell death or diffusion from the tumor sites as the likely causes for the drop in RLuc production following cell implantation.

The therapeutic effectiveness of hAMSCs was evaluated by comparing their capacity to inhibit U87 tumor growth by implanting the optimal 4∶1 (hAMSC to U87) proportion in mice brains, and treating with GCV or PBS. Such experiments showed that treatment with GCV of tumors containing hAMSCs reduced the number of tumor cells by a factor of 10^4^, relative to tumors without hAMSCs, also treated with GCV, or tumors with hAMSCs treated with PBS.

For the same tumor, by the end of the experiment, treatment with hAMSCs plus GCV had reduced tumor cell number to 0,12% of that at T = 0. However, in tumors without hAMSCs but also treated with GCV tumor cell number increased by 319 fold in the same period.

Macroscopic and laser confocal microscope examination of brain slices at the end of experiments corroborated BLI imaging results. While brains from mice with untreated mixed-cell tumors had large tumor masses containing green and red fluorescent cells, no macroscopic tumors and few green fluorescent cells were found in the brains of mice implanted with tumors plus therapeutic cells and treated with GCV.

In experiments better modeling a clinical situation, we show that implantation of therapeutic hAMSCs on preestablished glioblastoma U87 tumors and treatment with GCV also results in tumor growth inhibition that lasts as long as there were surviving therapeutic hAMSCs. Moreover, repeated inoculations of therapeutic hAMSCs resulted in a progressive reduction of tumor size and a significant extension of mice survival, relative to untreated controls.

Insight on the therapeutic mechanism mediated by hAMSCs was gained using a double label strategy that allows monitoring of changes in gene expression on a “per cell" basis. This was achieved by labeling of the same therapeutic cell that already expressed Rluc-RFP reporter constitutively, with a different Pluc-eGFP reporter regulated by the inducible human PECAM promoter. By co-implanting these cells with unlabelled U87 tumors and monitoring by BLI we could observe that within the first 7 days post implantation, a large proportion of the hAMSCs disappeared from the tumor site, either by death or dispersion. However, a subpopulation of the cells that expressed PECAM-promoter regulated luciferase remained in close association with the tumor. Moreover, in the tumor associated cell population, the ratio of PECAM-regulated to CMV-regulated luciferase activity increased with time reaching by day 7 a value 92 fold higher than that at implantation time. This results indicated that tumor associated hAMSCs were actively differentiating to the endothelial lineage. Independent analysis by fluorescence laser confocal microscopy of tumor slices revealed RFP and eGFP expressing hAMSCs with endothelial morphology in close association with tumor vascular structures. This latter result was further corroborated by positive staining of microvessel associated red fluorescent hAMSCs with an anti human PECAM antibody.

Association of bone marrow derived MSCs with vascular structures in tumors has been reported previously [29], [30]. However, the vascular associated cells were shown to express pericyte specific antigens while PECAM/CD31 is considered an endothelial specific marker. Both findings are not mutually exclusive, and in the absence of additional data, could be reconciled by considering that differentiation of mesenchymal stromal cells may depend on their tissue of origin as well as on the specific tumor environment in which they are implanted [31]. Our results suggest that GCV induced suicide of hAMSCs not only kills neighboring tumor cells by bystander effect but also by eliminating the associated vascular system that supplies oxygen and nutrients.

In conclusion, we demonstrate a general and versatile strategy to develop cell based tumor therapies and evaluate their effectiveness based on the use of chimeric bioluminescent and fluorescent reporters introduced in tumor and therapeutic cells, followed by analysis of their behavior by non invasive real time BLI and confocal microscopy.

With this strategy we were able to show that tTK expressing hAMSCs are very effective vehicles to deliver localized cytotoxic therapy to U87 glioblastomas and that hAMSCs within tumors differentiate to endothelial lineage cells that associate with vascular structures. Implantation of genetically modified hAMSCs may provide an effective procedure to eliminate residual tumor cells in surgical borders after tumor removal.

In future research we will use the above strategy to improve the preclinical significance of the therapy model. We will focus on evaluating procedures for therapeutic cell delivery, including the use of a biomaterial to protect and control the delivery of therapeutic cells implanted in tumors. To evaluate the relevance of the immune environment on the therapeutic effectiveness, we will use immune-competent mice implanted with singeneic glioma tumor model that will be treated with autologous therapeutic hAMSCs. Finally, we will seek to validate the generality of the therapeutic procedure using glioma tumors from human biopsies implanted in immunedefficient mice.

## Materials and Methods

### Vector constructs

Three lentiviral vectors constructs were used to label cells. hrl-mrfp-tk vector contains a chimeric trifunctional reporter comprising Rluc, monomeric red fluorescent protein (mRFP1) and a truncated version of the herpes simplex virus thymidine kinase coding sequences (sr39tk) regulated by the Cytomegalovirus promoter, was constructed [27]. pRRL-Luc-IRES-EGFP vector, containing the PLuc under control of the cytomegalovirus (CMV) promoter followed by an IRES to express an eGFP, was a kind donation from L. Alvarez-Vallina [32]. pLox:Pecam-Luc:eGFP vector that expresses chimeric Pluc and eGFP regulated by the endothelial specific promoter PECAM was constructed in our group. Lentiviral vector containing a fusion reporter comprising PLuc and eGFP, was obtained by polymerase chain reaction amplification and standard cloning procedures using the Pluc and eGFP genes from plasmid pGL4.10:PLuc (Promega Corporation, Madison, WI, USA) and pEGFP-N1 plasmid (Clontech Lab., Palo Alto, CA, USA). hPECAMp was kindly provided by Dr. Carmelo Bernabéu.

### Lentiviral particle production

Production of viral particles was performed using human embryonic kidney cells 293T (ATCC, CRL-11268™) grown in Dulbecco's Modified Eagle's Medium-high glucose (DMEM-hg) (Sigma, Steinheim, Germany), 10% heat-inactivated fetal bovine serum (FBS) (Sigma), 2 mM L-glutamine (Sigma), 50 units/ml penicillin/streptomycin (Sigma), and 2 mM HEPES. The day previous to transfection, 3×10^6^ cells were seeded on 10 cm^2^ poly-D-lysine (Sigma) treated plates. 6 µg of each lentiviral transfer vector, were mixed with 2 µg of viral envelope plasmid (pMD-G-VSV-G) and 4 µg of packaging construct (pCMV DR8.2) in 250 µl of 150 mM NaCl and then mixed with 48 µl of 1 mg/ml polyethylene amine (Polyscience, Warrington, PA, US) in 250 µl of 150 mM NaCl, and incubated at room temperature (RT) for 20 min. This DNA solution was then added drop wise to the plate containing the 293T cells plus medium, swirled gently and incubated for 16 h at 37°C with 5% CO*_2_*. The following day, the transfection solution was removed, the cells were rinsed with PBS 1X and medium without FBS was added to the cells. Following a 48 h incubation the supernatant was collected, centrifuged at 2000 rpm to remove cell debris, and filtered through a 0,45 µm low protein binding filter (Corning, Bath, UK).

### Cell lines and cell culture

hAMSCs were isolated from adipose tissue derived from cosmetic subdermal liposuctions, with patient consent, as described previously [26]. Liposuction samples were obtained after written informed consent by anonymous donors from Hospital de la Santa Creu i Sant Pau, Barcelona, Spain. Work with human samples was approved by written consent by the Ethical Committee of Clinical Investigation of Hospital Santa Creu i Sant Pau, Barcelona, Spain; and Bioethical Subcommittee of Superior Council of Scientific Research. Briefly, lipoaspirate was suspended in 1X collagenase type I (Invitrogen, Carlsbad, CA) solution and incubated at 37°C and inactivated by addition of DMEM+10% FBS. hAMSC were isolated by plastic adherence technique. hAMSCs were grown in DMEM-hg with 20% FBS (Hyclone, Logan, UT), 2 mM L-glutamine (Sigma) and 50 units/ml penicillin/strepotomycin (Sigma), expanded for until 70% of confluence and infected with either the hrl-mrfp-tk, or with both, first with the hrl-mrfp-tk and then with the pLox:Pecam-Luc:eGFP viral vector stocks, using 10 µg/ml polybrene (Sigma) and incubated for 24 to 48 hours (MOI = 20). Human glioma cells U87 (ATCC, HTB-14), were grown in nutrient mixture DMEM/Hams F12 HAM containing 10% heat-inactivated FBS (Sigma), 2 mM L-glutamine (Sigma) and 50units/ml penicillin/strepotomycin (Sigma). U87 were infected, as described, with pRRL-Pluc-IRES-eGFP viral stock (MOI = 20) to obtain Pluc-G-U87, as described above. Expression of fluorescent proteins was used to select positively transduced cells.

### Cell proliferation assays

Quadruplicates of 3×10^3^ Rluc-R-tTK-hAMSC or untransduced hAMSCs an 5×10^3^ Pluc-G-U87 cells were plated into 96-well plates and incubated overnight at 37°C. Culture medium was replaced with medium containing 4 µg/ml GCV 24 h later (T = 0). Cell proliferation was evaluated spectrophotometrically at 490 nm, using the CellTiter 96 Aqueous One Solution Cell Proliferation Assay (Promega) at days 0, 5 and 9. Results were expressed as the percentage of proliferation, where the proliferation of cells in culture medium at day 0 was set to 100%. Mixed cell cultures containing 10^4^ Pluc-G-U87 with several proportions of Rluc-R-tTK-hAMSC were seeded on 48-well plates and analyzed by BLI at days 0 (the onset of GCV treatment), 5 and 9, with changes of medium every 3 days. Medium was changed every 3 days.

### BLI determination of luciferase activity

#### *In vitro* cultures

For BLI of tissue culture plates, medium was removed from the wells, the wells were rinsed twice with PBS 1×, and imaged immediately following addition of 100 µl/well of Pluc or Rluc substrate stock reagent (Caliper, Hopkinton, MA, US and Prolume, Pinetop, AZ, US). Imaging of PLuc and Rluc activities was performed in consecutive days.

For imaging, plates are placed in the detection chamber of a high-efficiency ORCA-2BT Imaging System (Hamamtsu Photonics, Hamamatsu City, Japan) provided with a C4742-98-LWG-MOD camera fitted with a 512×512 pixel charge couple device (CCD) cooled at −80°C. Images were acquired during 1 min using 1×1 array (binning 1×1), and in order to register the position of the light signal, an additional image was obtained using a white light from a lamp in the detection chamber. Light events were calculated using the Wasabi image analysis software and expressed as PHC after discounting the background of wells without cells. The net number of PHCs in the area of interest was calculated using the formula: PHCs = (total number of PHCs in the area of interest)−[(number of pixels in the area of interest)×(average background PHCs per pixel)].

Pseudo color images were generated using arbitrary color bars representing standard light intensity levels for Pluc (blue: lowest; red: highest) and for Rluc (black: lowest; blue: highest).

#### *In vivo* bioluminescent imaging

*In vivo* BLI of engrafted SCID mice was performed as described previously [26]. Briefly, mice were anesthetized i.p. and then injected i.p. with 150 µl of luciferin (Caliper) (16.7 mg/ml in saline) or through tail vein with 25 µl of benzyl coelenterazine (1 mg/ml in 50/50 propilenglycol/ethanol) (Prolume) diluted in 125 µl of water. When BLI was performed after the surgery no anesthesia was required. Animals were placed in the detection chamber of the high efficiency ORCA-2BT Imaging System and images acquired from the dorsal direction during a 5 min period. A second image of the animal was obtained using a white-light source inside the detection chamber, to register the position of the luminescence signal. To increase detection sensitivity, the readout noise of the recorded signal was reduced by adding together the light events registered by arrays of 8×8 adjacent pixels that are read simultaneously (binning 8×8) of the camera CCD. Mice were imaged weekly during experiments. Quantification and analysis of photons recorded in images was done using the Wasabi image analysis software (Hamamatsu Photonics) as described above.

In order to establish the correlation between the number of transplanted cells and the amount of emitted light, standard curves were generated by imaging either 1×10^4^, 2,5×10^4^, 5×10^4^ and 1×10^5^ Pluc expressing cells or 1×10^4^, 3×10^4^, 5×10^4^ and 1.5×10^5^ Rluc expressing cells, stereotactically injected in the brain (0.6 mm posterior, 2 mm lateral and 2.75 mm depth respect bregma). Light measurements were expressed as PHCs following subtraction of background as previously described. Data were represented as the number of PHCs versus number of grafted cells.

### Animal experiments

Adult 6–8 weeks old SCID mice were purchased from (Charles Rivers, Wilmington, MA, USA) and kept under pathogen-free conditions in laminar flow boxes. Animal maintenance and experiments were performed in accordance with established guidelines of Catalan Government and protocol num. 4565 approved by Direcció General del Medi Natural, Generalitat de Catalunya. Animals were anesthetized by i.p. injections of xylazine (Henry Schein, Melville, NY,USA) 3.3 mg/kg and ketamine (Merial, Duluth, GA, USA) 100 mg/kg. Subsequently mice were mounted in a stereotactic frame (Stoelting, Wood Dale, IL, U.S.A.) and their heads were secured using a nose clamp and two ear bars, and a skin flap was lifted to expose the skull surface and further anesthetized, by injection of fentanil (Kernpharma, Barcelona, Spain). For stereotactic cell implantation, a burr hole was drilled and the cell suspensions was injected at a 250 nl/min rate using a Hamilton syringe series 700 at the previously indicated coordinates. The injections were delivered at a rate of 0.25 µl/min, and the needle was slowly withdrawn after an additional 5 minutes. The scalp was closed by suture and the animals were placed in individual recovery cages and supplied with buprenorfine (Buprex, Schering Plough SA, Madrid, Spain) in the drinking water. GCV sodium (Cymevene, Roche, Basel, Switzerland) was injected i.p. daily at a dose of 100 mg/kg [33], [34].Control animals were inoculated with the same volume of PBS.

### Histology

Brain from sacrificed mice were harvested, washed with physiological serum and fixed with paraformaldehyde during 48 hours. Brains were then washed with PBS 1×, embedded in OCT, sliced in 20 µm sections and mounted on glass slides. Hoechst staining was performed for detection of cell nuclei. For macroscopic view analysis 20 µm brain sections were stained with Hematoxylin and Eosin (HE). Immunohistochemical detection of endothelial cells was performed on 10 µm thick section using a primary hPECAM-1 (4 µg/ml) (Abcam, Cambridge, UK). Secondary antibody conjugated with Cy5 (3 µg/ml) (Jackson ImmunoResearch, Suffolk, UK) was applied and sections counterstained with Bisbenzimide (Hoechst, Sigma).

### Fluorescence angiography and Laser Confocal microscope observation of brain implanted hAMSCs

For tumor microvessel imaging, mice were anesthetized and injected trough the lateral tail vein with 200 µl (10 mg/ml) of a high molecular weight (2,000,000 MW) FITC-conjugated dextran Sigma (St Louis, MO, US). Ten minutes after the injection mice were sacrificed and brains were retrieved and fixed in formalin solution 10% (Sigma) during 24 hours. The fixed brains were sliced and analyzed for microvessel formation and presence of fluorescent hAMSCs using confocal laser scanning microscopy (Leica TS1 SP2).

#### Flow citometry

Supporting file Methods S1.

### Statistical analysis

Student's unpaired two-tailed t-test was used for comparison between groups. Descriptive statistics were performed with SPSS Statistics (15.0.1 version, SPSS Inc., Chicago, IL). Data are presented as mean ± SEM and considered significant at p<0.05 or p<0.01.

## Supporting Information

Figure S1 **Characterisation of transduced and untransduced hAMSC.** (**A**) Flow cytometric analysis of hAMSCs (top) and RLuc-R-tTK-hAMSCs (below) using antibodies against CD 105, CD44, CD29, CD90, CD73, CD106, CD34, CD45 showed no difference in marker expression. (**B**) Proliferation rate of transduced and untransduced MSCs was evaluated spectrophotometrically by standard 3-(4-5-dimethyl-2-yl)-5-(3-carboxymethoxyphenyl)-2-(4-sulfophenyl)-2H-tetrazolium salt (MTS) assay and expressed as percentage of cell proliferation (respect day 0).
(TIF)

Figure S2 **Cell sensitivity to GCV.** RLuc-R-tTK-hAMSC, hAMSC and U87 were cultured in medium containing GCV at indicated concentrations (0 µg/ml, 2 µg/ml, 4 µg/ml and 8 µg/ml). Cell viability was evaluated spectrophotometrically by standard MTS assay and expressed as percentage of cell proliferation (respect day 0) * P<0,001 n = 4 for each group. (**A**) RLuc-R-tTK-hAMSC proliferation rate is sensitive to GCV dose. At day 5 there is a linear correlation between GCV concentration and cell death. (**B**,**C**) hAMSCs and U87 proliferate without significant change even in the presence of high concentrations of GCV confirming its low toxicity.
(TIF)

Figure S3 ***In vivo*** **monitorization of tumor progression in hAMSCs mediated therapy**. Pseudo-color images from 1∶4+GCV (**A**), 1∶4+PBS (**B**) and 1∶0+GCV (**C**) groups were acquired once per week. Luciferase images are superimposed on black and white with images of the same mouse. U: tumor unrelated death; T: tumor related death.
(TIF)

Methods S1 **Flow cytometry analysis of hAMSC markers.** hAMSC and RLuc-R-tTK-hAMSC suspended in phosphate-buffered saline plus 1% bovine serum albumin and incubated with mouse anti-human cd90-PE (BD Bioscience); mouse anti-human cd34-PE (ab-cam); mouse anti-human cd105-PE (BD Bioscience); mouse anti human cd 106-PE (BD, Bioscience); mouse anti-human cd73-PE (BD, Bioscience); mouse anti-human cd29 (BD, Bioscience); mouse anti-human cd45-FITC (BD, Bioscience); mouse anti-human cd44-PE(BD, Bioscience).Unspecific binding was assessed by isotype control mouse IgG2α-PE (BD, Biosciences), mouse IgG1κ-PE (BD, Bioscience) and mouse IgG1κ-FITC (BD, Bioscience). Antibody-FITC and PI binding was analyzed by FACS in an EPICS XL™ Flow Cytometer.
(DOC)
