# Supplementary figures and images for: Glioblastoma Therapy with Cytotoxic Mesenchymal Stromal Cells Optimized by Bioluminescence Imaging of Tumor and Therapeutic Cell Response
Source: PLoS One. 2012 Apr 17;7(4):e35148. doi: 10.1371/journal.pone.0035148 (PMC3328467; doi:10.1371/journal.pone.0035148)

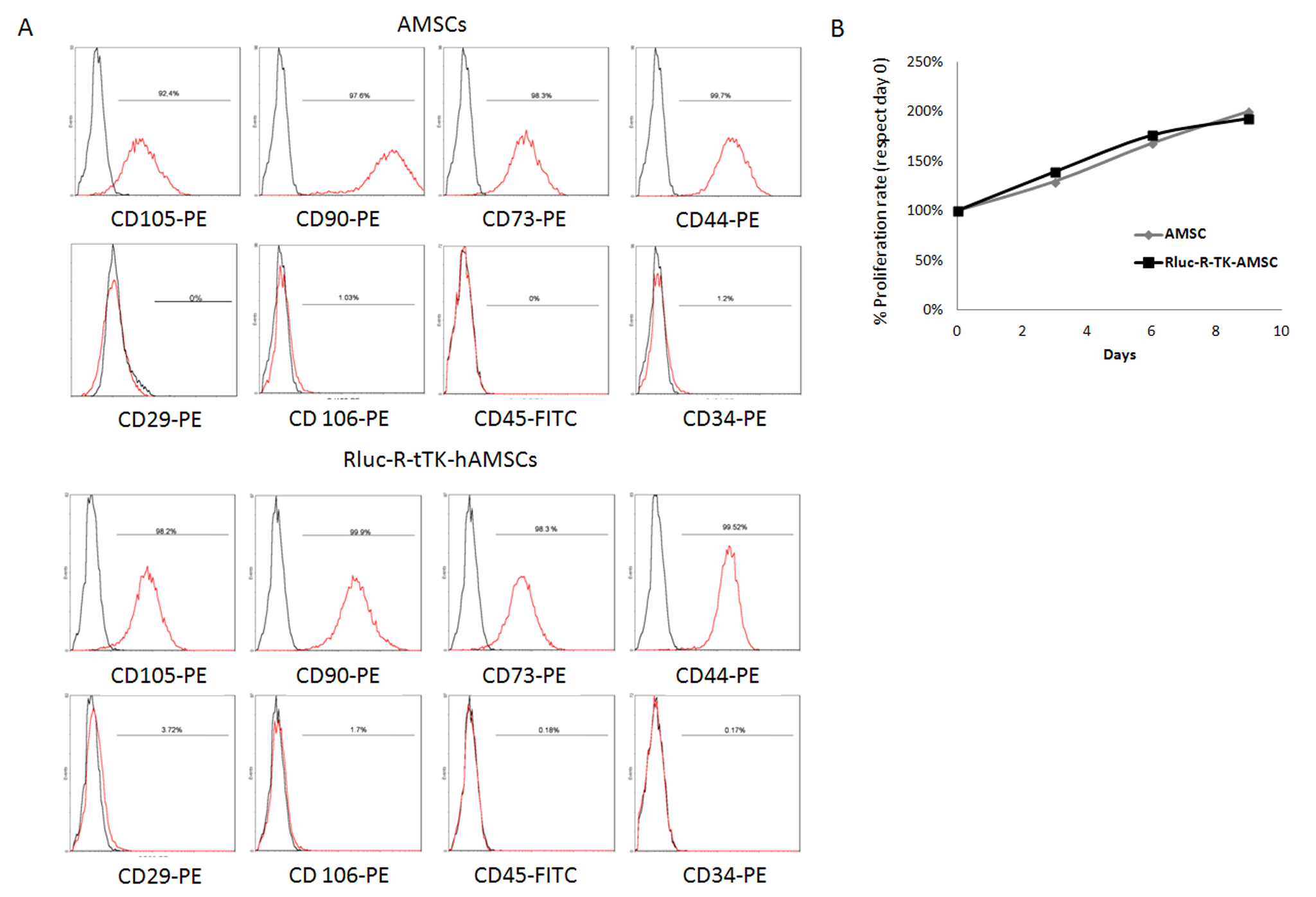

Supplement: Figure S1 — Characterisation of transduced and untransduced hAMSC. (A) Flow cytometric analysis of hAMSCs (top) and RLuc-R-tTK-hAMSCs (below) using antibodies against CD 105, CD44, CD29, CD90, CD73, CD106, CD34, CD45 showed no difference in marker expression. (B) Proliferation rate of transduced and untransduced MSCs was evaluated spectrophotometrically by standard 3-(4-5-dimethyl-2-yl)-5-(3-carboxymethoxyphenyl)-2-(4-sulfophenyl)-2H-tetrazolium salt (MTS) assay and expressed as percentage of cell proliferation (respect day 0). (TIF) [file pone.0035148.s001.tif]

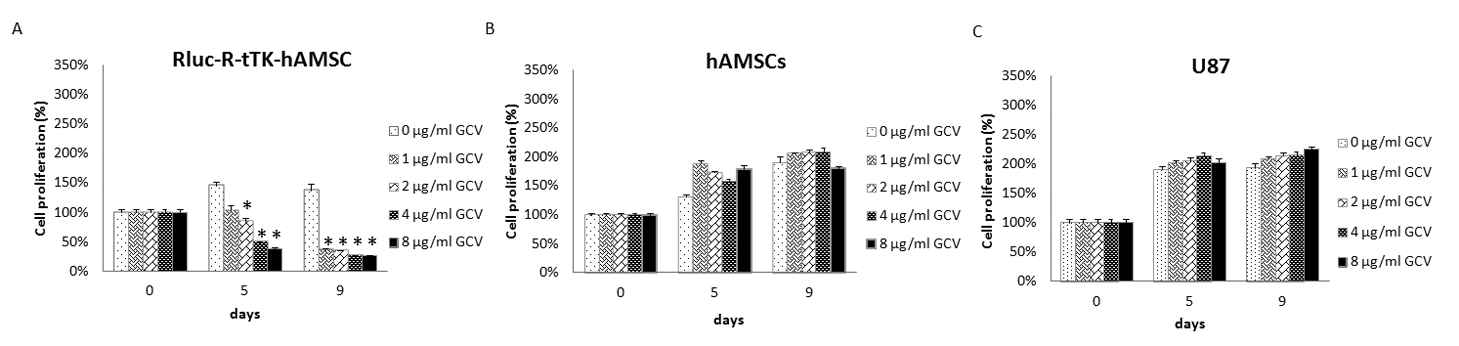

Supplement: Figure S2 — Cell sensitivity to GCV. RLuc-R-tTK-hAMSC, hAMSC and U87 were cultured in medium containing GCV at indicated concentrations (0 µg/ml, 2 µg/ml, 4 µg/ml and 8 µg/ml). Cell viability was evaluated spectrophotometrically by standard MTS assay and expressed as percentage of cell proliferation (respect day 0) * P<0,001 n = 4 for each group. (A) RLuc-R-tTK-hAMSC proliferation rate is sensitive to GCV dose. At day 5 there is a linear correlation between GCV concentration and cell death. (B,C) hAMSCs and U87 proliferate without significant change even in the presence of high concentrations of GCV confirming its low toxicity. (TIF) [file pone.0035148.s002.tif]

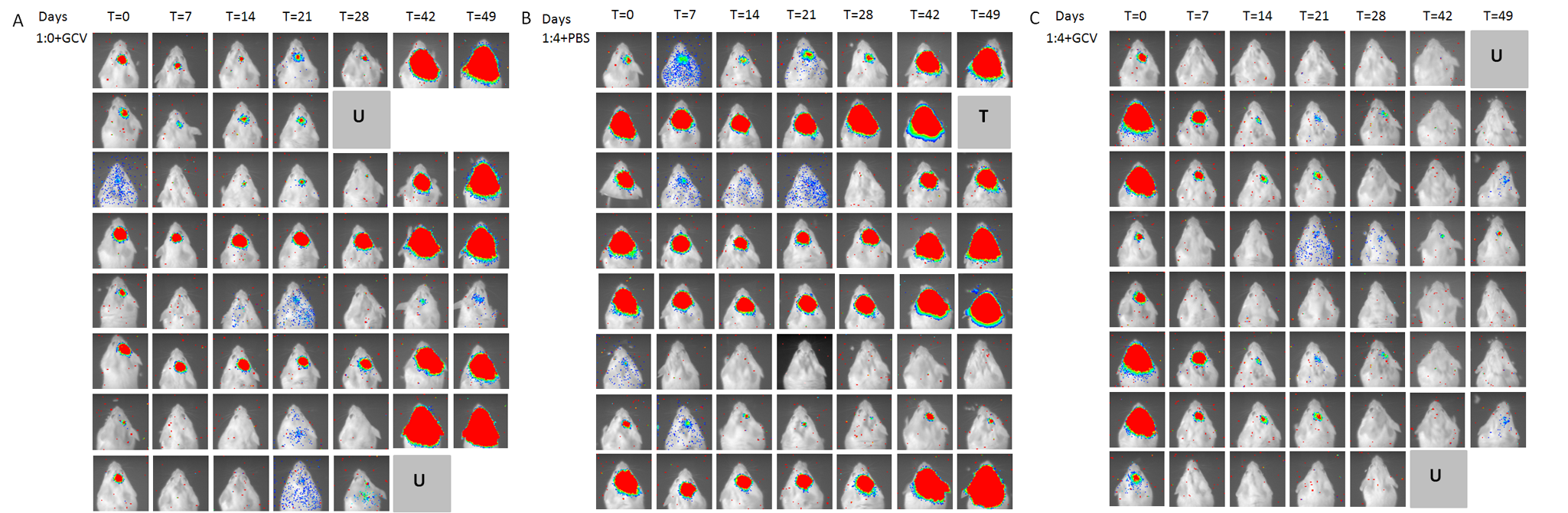

Supplement: Figure S3 — In vivo monitorization of tumor progression in hAMSCs mediated therapy. Pseudo-color images from 1∶4+GCV (A), 1∶4+PBS (B) and 1∶0+GCV (C) groups were acquired once per week. Luciferase images are superimposed on black and white with images of the same mouse. U: tumor unrelated death; T: tumor related death. (TIF) [file pone.0035148.s003.tif]
