# Supplementary material for: Glioblastoma Therapy with Cytotoxic Mesenchymal Stromal Cells Optimized by Bioluminescence Imaging of Tumor and Therapeutic Cell Response
Source: PLoS One. 2012 Apr 17;7(4):e35148. doi: 10.1371/journal.pone.0035148 (PMC3328467; doi:10.1371/journal.pone.0035148)
Supplement: Methods S1 — Flow cytometry analysis of hAMSC markers. hAMSC and RLuc-R-tTK-hAMSC suspended in phosphate-buffered saline plus 1% bovine serum albumin and incubated with mouse anti-human cd90-PE (BD Bioscience); mouse anti-human cd34-PE (ab-cam); mouse anti-human cd105-PE (BD Bioscience); mouse anti human cd 106-PE (BD, Bioscience); mouse anti-human cd73-PE (BD, Bioscience); mouse anti-human cd29 (BD, Bioscience); mouse anti-human cd45-FITC (BD, Bioscience); mouse anti-human cd44-PE(BD, Bioscience).Unspecific binding was assessed by isotype control mouse IgG2α-PE (BD, Biosciences), mouse IgG1κ-PE (BD, Bioscience) and mouse IgG1κ-FITC (BD, Bioscience). Antibody-FITC and PI binding was analyzed by FACS in an EPICS XL™ Flow Cytometer. (DOC) [file pone.0035148.s004.doc]

**SUPPORTING METHODS**

**Flow cytometry**

hAMSC and RLuc-R-tTK-hAMSC suspended in phosphate-buffered saline plus 1% bovine serum albumin and incubated with mouse anti-human cd90-PE (BD Bioscience); mouse anti-human cd34-PE (ab-cam); mouse anti-human cd105-PE (BD Bioscience); mouse anti human cd 106-PE (BD, Bioscience); mouse anti-human cd73-PE (BD, Bioscience); mouse anti-human cd29 (BD, Bioscience); mouse anti-human cd45-FITC (BD, Bioscience); mouse anti-human cd44-PE(BD, Bioscience).Unspecific binding was assessed by isotype control mouse IgG2α-PE (BD, Biosciences), mouse IgG1κ-PE (BD, Bioscience) and mouse IgG1κ-FITC (BD, Bioscience). Antibody-FITC and PI binding was analyzed by FACS in an EPICS XL^TM^ Flow Cytometer.
